# Secretory function in subplate neurons during cortical development

**DOI:** 10.3389/fnins.2015.00100

**Published:** 2015-03-26

**Authors:** Shinichi Kondo, Hannah Al-Hasani, Anna Hoerder-Suabedissen, Wei Zhi Wang, Zoltán Molnár

**Affiliations:** Department of Physiology, Anatomy and Genetics, University of OxfordOxford, UK

**Keywords:** subplate neurons, rough endoplasmic reticulum, ultrastructural analysis, ER stress condition, cerebral cortex, neuroserpin, *serpini1*

## Abstract

Subplate cells are among the first generated neurons in the mammalian cerebral cortex and have been implicated in the establishment of cortical wiring. In rodents some subplate neurons persist into adulthood. Here we would like to highlight several converging findings which suggest a novel secretory function of subplate neurons during cortical development. Throughout the postnatal period in rodents, subplate neurons have highly developed rough endoplasmic reticulum (ER) and are under an ER stress condition. By comparing gene expression between subplate and layer 6, we found that several genes encoding secreted proteins are highly expressed in subplate neurons. One of these secreted proteins, neuroserpin, encoded by the *serpini1* gene, is localized to the ER in subplate cells. We propose that subplate might influence cortical circuit formation through a transient secretory function.

## Introduction

All eukaryotic cells contain a discernible amount of rough endoplasmic reticulum (ER) because it is needed for the synthesis of plasma membrane proteins and proteins of the extracellular matrix (Depierre and Dallner, 1975). Rough ER is particularly abundant in cells that are specialized to produce secreted proteins. For example, plasma cells produce antibodies, which circulate in the bloodstream, and pancreatic acinar cells synthesize digestive enzymes, which are transported to the intestine. In both types of cells, a large part of the cytosol is filled with rough ER. When cells synthesize secretory proteins in amounts that exceed the capacity of the folding apparatus, unfolded proteins accumulate in the rough ER. To alleviate such an overstretched functional state, eukaryotic cells activate a series of self-defense mechanisms referred to collectively as the ER stress response (also called the unfolded protein response) (Schroder and Kaufman, 2005). ER stress response is especially observed physiologically for dedicated secretory cells, such as plasma cells, pancreatic acinar cells, and pancreatic beta cells, where high levels of secreted protein synthesis require a highly evolved mechanism to properly fold, process and secrete them (Wu and Kaufman, 2006; Kondo et al., 2011).

In neurons, when stained with basic aniline dyes (toluidine blue, thionine, or cresyl violet), rough ER appears under the light microscope as a basophilic granular area called Nissl substance. The amount of Nissl substance varies according to neuronal type and functional state. It is particularly abundant in large nerve cells, especially motor neurons (Einarson, 1935). Under different physiological conditions, and in certain pathological states, Nissl substance changes it's appearance. However, the mechanism underlying this change remains unclear. Interestingly, it has been reported that malfunction of the ER stress response can result in neurodegenerative disorders (Paschen and Mengesdorf, 2005), but it remains unclear whether ER stress response occurs physiologically in neurons *in vivo*.

Subplate neurons are among the first generated neurons in the mammalian cerebral cortex and are important in establishing correct intra- and extra-cortical connectivity. Transient neurons of the subplate are considered to be instrumental in the development of the cortex and in the establisment of corticothalamic and thalamocortical connections (Kostovic and Rakic, 1990; Allendoerfer and Shatz, 1994; Kanold and Luhmann, 2010; Hoerder-Suabedissen and Molnár, 2015). While, in most mammalian species including primates, the majority of subplate neurons are lost in the development of the cortex (Kostovic and Rakic, 1980), a large proportion of the subplate persists into adulthood in rodents (Woo et al., 1991). Although there has been a huge progress in understanding the role of subplate neurons in establishing cortical circuits, additional functions of subplate neurons have not been clarified.

In this report, we would like to propose a novel secretory function for subplate neurons. We performed morphological analysis with special reference to the rough ER. To examine the functional state, we used Nissl stain and immunohistochemistry for ER stress proteins [Binding immunoglobulin protein (BiP) also known as 78 kDa glucose-regulated protein (GRP-78) or heat shock 70 kDa protein 5 (HSPA5)], and electron microscopic analysis. We also analyzed published subplate gene expression profiles (Hoerder-Suabedissen et al., 2009, 2013; Oeschger et al., 2011) for genes encoding secreted proteins and validated the expression of candidate genes by immunohistochemistry.

## Materials and methods

### Animals and tissue preparation

All animal experiments were approved by a local ethical review committee and conducted in accordance with personal and project licenses under the UK Animals (Scientific Procedures) Act (1986). For light microscopy analysis, three P8 and three adult C57BL/6 mice were anesthetized using pentobarbitone (Euthatal 150 mg/kg intraperitoneally; Merial Animal Health Ltd, Harlow, UK) and perfused through the heart with 4% paraformaldehyde (PFA; TAAB, Reading, UK) in phosphate-buffered saline (PBS, 0.1 M; pH 7.4). The brains were removed, dissected and fixed in the same fixative for 24 h at 4°C. For electron microscopy analysis, three Wistar rats at P8 were anesthetized using pentobarbitone and perfused through the heart with 4% PFA with 1% glutaraldehyde in 0.1M-phosphate buffer (PB; pH7.4). The brains were removed, dissected, and fixed in the same fixative for 2 days at 4°C.

### Histological processing

Fixed mouse brains were embedded in paraffin. Serial coronal sections were cut at a thickness of 8 μm and divided into two series. One set was used for Nissl staining, and another was prepared for immunohistochemistry. For Nissl staining, sections were stained with 0.1% cresyl violet solution. For immunohistochemistry, the section were incubated in 2% normal goat serum (NGS) diluted in Tris-buffered saline (TBS; 50 mM Tris buffer, 0.09% NaCl, pH 7.4) for blocking, and then incubated for 2 h at room temperature (RT) with mouse anti-KDEL antibody (1:500, Abcam) as anti-BiP (Okiyoneda et al., 2004) and rabbit anti-neuroserpin antibody (1:200, Abcam) in 1% NGS diluted in TBS. Following several washes, anti-mouse-AlexaFluor488 antibody (1:500, Molecular Probes) and anti-rabbit-AlexaFluor546 antibody (1:500, Molecular Probes) diluted in 1% NGS in TBS were applied for 2 h at RT. The sections were imaged using an epifluorescent microscope (DMR; Leica Microsystems). We selected P8 for our analysis based on the data obtained from our microarray-based gene expression analysis (Hoerder-Suabedissen et al., 2013; https://dpag.cloudant.com/subplate-atlas/_design/subplate-atlas/index.html).

### Electron microscopy processing

Fixed rat brains were rinsed in 0.1M-PB (pH7.4) and post-fixed with osmium tetraoxide. Once the tissue was osmicated it was then rinsed with 0.1M PB followed by dehydration through graded alcohols and placed in propylene oxide. The tissue was prepared for sectioning by placeing it in propylene oxide:Epon Araldite 1:1 overnight, followed by Epon Araldite for a further night, before being embedded in fresh Araldite and placed at 60°C for 48 h to harden fully. Semi-thin (1 μm) sections were stained with 1% toluidine blue in order to select suitable areas for transmission electron microscopy. Sections were mounted on copper grids, stained with uranyl acetate (5% UA in 50% alcohol) and Reynolds lead citrate, and examined in a JEOL EM15007 electron microscope.

### Subplate dissection and RNA isolation

For detailed description of the microarray experiments identifying subplate enriched genes please see Hoerder-Suabedissen et al. (2009). Briefly, P8 mouse brains were sectioned into 150 μm parasagittal sections and thin strips of anterior subplate and adjacent layer 6 and posterior subplate and layer 6 were dissected out under visual guidance using transillumination on a dissecting microscope. 8 fragments of each tissue type for each brain were included and pooled the fragments of 4 littermates per replicate. A total of 4 biological replicates were collected for each location. Total RNA was isolated using the RNeasy Micro kit (Qiagen, Crawley, UK) following the manufacturer's instructions. The quality and RNA integrity were assessed on a BioAnalyzer; all samples had a RNA Integrity Number 8 (Agilent Laboratories, Stockport, UK). Labeled cRNA for hybridization was generated with the Affymetrix “2 Cycle Target Labeling and Control” kit (Affymetrix, High Wycombe, UK) and MEGAscript T7 polymerase (Ambion) according to the manufacturer's instructions. Labeled anti-sense cRNA was fragmented and the distribution of fragment lengths was measured using a BioAnalyzer (Agilent). Labeled and fragmented cRNA was hybridized to the Affymetrix 430 2.0 whole mouse genome microarray (Affymetrix). A total of 16 chips were used, all from the same batch. Chips were processed on an Affymetrix GeneChip Fluidics Station 450 and Scanner 3000.

### Microarray analysis

For detailed description of the normalization, clustering, statistical analysis on the microarray data please see Hoerder-Suabedissen et al. (2009). Briefly: arrays were Robust Multi-Array (RMA) normalized, and differentially expressed genes were identified using a paired *t*-test with a cut off *p*-value < 0.05 (no multiple testing correction) and a >1.5 fold-change difference between any 2 comparisons. Longer lists of differentially expressed genes (>1.5-fold difference, *p* < 0.05) were generated from RMA taking GC content into account (GCRMA) normalized data.

### Computational gene ontology analysis

To classify cellular distribution of the proteins, gene ontology (GO) analysis was performed using GO_Full ontology (http://www.geneontology.org). The list of P8 subplate enriched genes was examined specifically for secretory genes. A list of genes with products localized in the extracellular space was generated and the examples were selected because they had the highest (first 4 on the list) expression levels in absolute mRNA volume (Table 1 and https://molnar.dpag.ox.ac.uk/subplate/).

**Table 1:** **This table lists some of the genes expressed at a high level in subplate neurons which also localize to the extracellular space**.

**Gene name**	**Cellular localization**	**Probe set**	**Anterior fold change**	**Posterior fold change**
Connective tissue growth factor (CTGF)	extracellular space	1416953_at	11.2	14.9
Neuron-specific serine protease inhibitor (neuroserpin)	extracellular space	1448443_at	2.4	1.7
Neuronal pentraxin 1(Nptx1)	extracellular space	1434877_at	2.2	2.7
Insulin-like growth factor binding protein 5 (IGFBP-5)	extracellular space	1452114_s_at	2.5	2.1

*Affymetrix probe set IDs are given in the probe set column. Fold-changes reflect the difference in gene expression levels between subplate and layer 6a at P8 in anterior (S1) and posterior (V1) regions, and are calculated as mean fold-changes across all four replicates. Data from Hoerder-Suabedissen et al. (2009) and (2013). Additional data can be found at https://molnar.dpag.ox.ac.uk/subplate/*.

## Results

### Subplate neurons in P8 mouse brain have extensive Nissl substance

**Figure 1:**
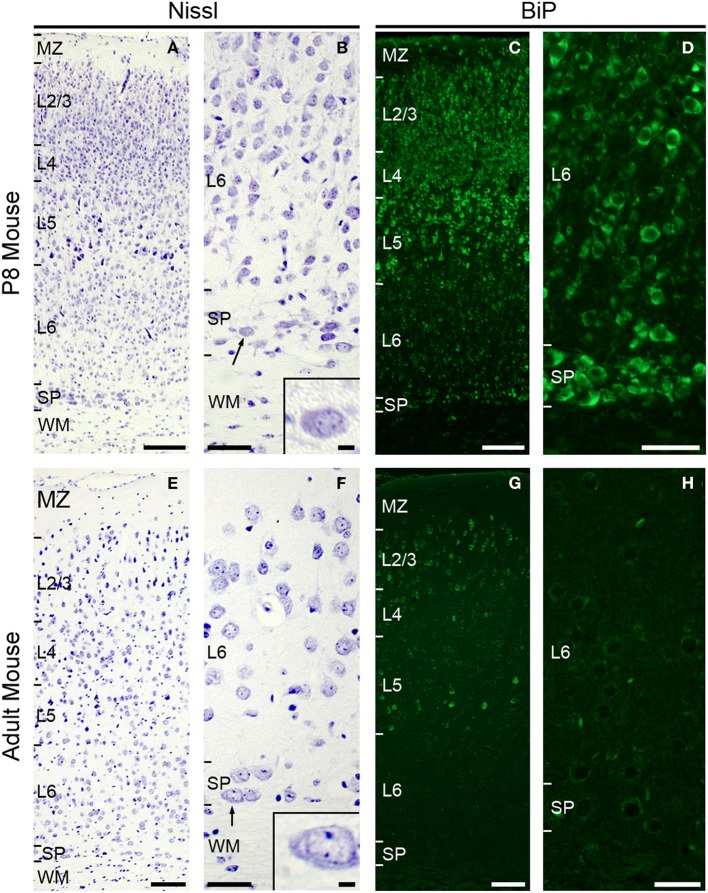
**Subplate neurons have large cytoplasm with large amounts of Nissl substance and are under ER stress condition at P8**. Nissl staining in coronal section of P8 **(A,B)** and adult **(E,F)** mouse brain. Note, subplate neurons (and some layer 5 and 2–3 neurons) have voluminous cytoplasm with large amounts of Nissl substance (arrow and inset, **B**) at P8. Subplate neurons in adult mouse have relatively small cytoplasm (arrow and inset, **F**). Immunohistochemistry for anti-KDEL antibody, which recognizes BiP/GRP78 (Okiyoneda et al., 2004), in coronal section of P8 mouse **(C,D)**. Layer 5, layer 2–3, some layer 6, and subplate neurons express strong BiP immunoreactivity. Immunohistochemistry for anti-KDEL in coronal section of adult mouse **(G,H)**. Scale bars: 200 μm **(A,C,E,G)**, 50 μm **(B,D,F,H)**, 10 μm (inset in **B** and **F**).

To characterize the morphology and the functional state of subplate neurons in postnatal and adult rodents, we analyzed Nissl stained coronal sections of P8 mouse brains (Figures 1A,B) and adult mouse brains (Figures 1E,F). At P8, extensive Nissl substance was detected in the large pyramidal cells of layer 5. Plentiful Nissl substance was also observed in layer 2/3 and subplate neurons (Figure 1A), suggesting that these neurons produce a large amount of proteins at P8. The morphological features of subplate neurons (Figure 1B) are surprisingly similar to those of plasma cells, which also have a large, ovoid cell body with non-central distribution of nucleus and basophilic cytoplasm due to their richness in rough ER (Bloom and Fawcett, 1968).

In the adult mouse brain, abundant Nissl substance was detected in pyramidal cells of layer 2/3 and layer 5 (Figure 1E), while the cell bodies of subplate neurons appear small and weakly stained (Figure 1F).

### Endoplasmic reticulum stress occurs in mouse subplate neurons at P8

To investigate whether the amount of Nissl substance correlates with ER stress, we next examined the expression level of the ER stress marker protein BiP (also called GRP78) (Okiyoneda et al., 2004; Kondo et al., 2005, 2012; Schroder and Kaufman, 2005; Wu and Kaufman, 2006). The induction of the ER chaperone protein BiP, which is required for the proper folding and assembly of secretory proteins, is a major ER stress response. BiP is up-regulated under stress conditions, such as glucose deprivation, hypoxia, or the presence of toxic agents (Lee, 2001). Immunohistochemical analysis using the anti-KDEL antibody, which recognizes BiP (Okiyoneda et al., 2004), showed strong expression of the BiP protein in pyramidal cells of layer 2/3 and layer 5 and subplate neurons in the P8 mouse brain (Figures 1C,D). In adult brains, although pyramidal cells in layer 2/3 and 5 continue to express massive amounts of BiP protein, we could not detect BiP expression in subplate/layer 6b neurons (Figures 1G,H). These results suggest that ER stress occurs in subplate neurons at early postnatal, but not or much less in adult ages.

### Subplate neurons have highly developed rough ER during development

**Figure 2:**
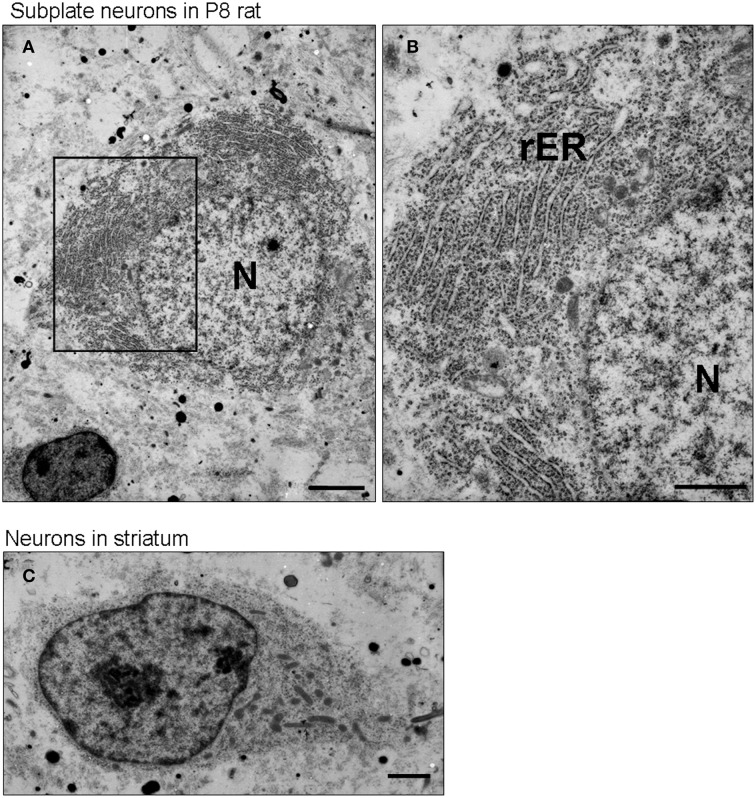
**Subplate neurons have a well-developed rough endoplasmic reticulum**. Transmission electron microscopic image of a subplate neuron of P8 rat brains **(A,B)**. Note, the large amounts of rough ER (rER) in the subplate neurons. The chromatin in the nucleus (N) is not strongly condensed. For comparison, see the transmission electron microscopic image of a neuron in striatum of P8 rat brains **(C)**, in which cells display much less rER. Scale bars: 2 μm **(A)**, 1 μm **(B,C)**.

To confirm directly whether subplate neurons in postnatal rodents have well developed rough ER, we carried out ultrastructural analysis of P8 rat brains using electron microscopy (Figure 2). Electron micrographs of subplate neurons showed an abundance of rough ER (Figures 2A,B) compared to either neurons in the striatum (Figure 2C) or pyramidal cells in layer 5 during postnatal period (Miller and Peters, 1981) or in adult (Parnavelas and Lieberman, 1979). The chromatin in the nucleus of subplate neurons is not strongly aggregated at P8, suggesting that high levels of mRNAs are being produced. The presence of a well-developed rough ER in subplate neurons during the postnatal period suggests an active protein production function for these cells.

### Subplate neurons in P8 mouse brain express secreted proteins

**Figure 3:**
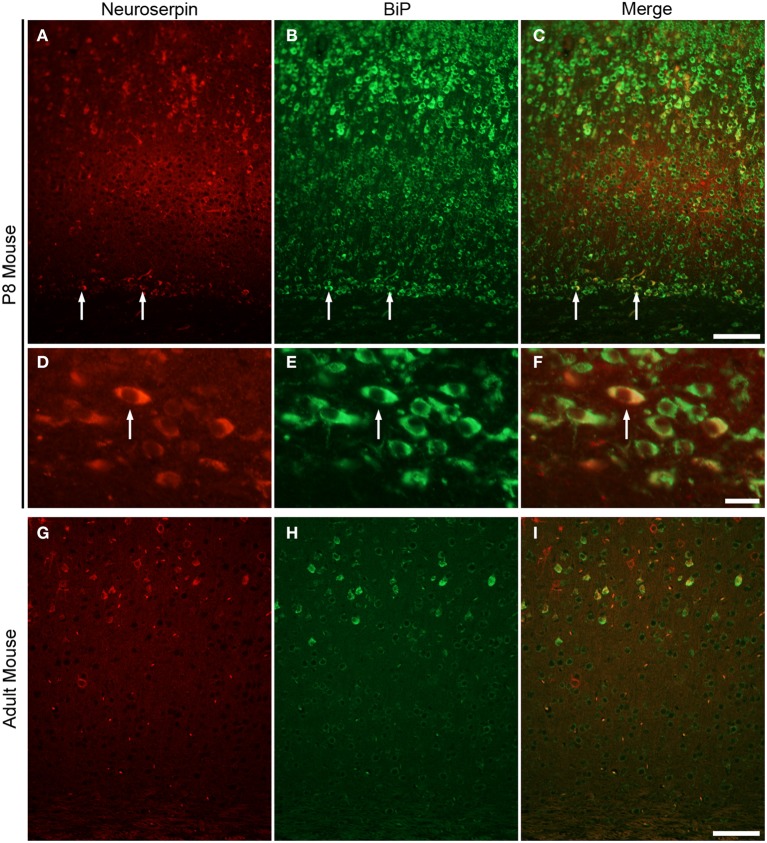
**Subplate neurons in P8 mouse brain strongly express neuroserpin**. Immunohistochemistry for anti-neuroserpin **(A)** and anti-KDEL **(B)** and their correlation **(C)** in coronal section of P8 mouse. Note, co-localization of neuroserpin and BiP in subplate neurons (arrows, **C**; cell in **D–F**). Immunohistochemistry for anti-neuroserpin **(D)** and anti-KDEL **(H)** in coronal section of adult mouse. Neither neuroserpin nor BiP is strongly expressed in the adult subplate. Scale bars: 100 μm (**A–C**, **G–I**), 10 μm (**D–F**).

The plasma cell has a well-developed rough ER to be able to synthetise and secret massive amounts of antibodies. Because of their very similar subcellular morphology (Bloom and Fawcett, 1968), we postulate that subplate neurons also a secretory function. To elucidate this possibility we analyzed the gene expression profile for P8 mouse subplate generated from a microarray comparison on subplate and layer 6a tissue samples (Hoerder-Suabedissen et al., 2009, 2013). Comparing gene expression in the subplate with the adjacent layer 6a in somatosensory and visual cortices, we identified 601 probe sets (corresponding to 383 genes and hypothetical genes) that were expressed at a higher (at least 1.5-fold) level in the subplate compared with layer 6 in both comparisons (Hoerder-Suabedissen et al., 2009). Gene ontology (GO) analysis for cellular localization was performed on this list. Table 1 shows some selected examples of genes that encode extracellular proteins and have the highest four expression levels in absolute mRNA volume. Gene expression of these four genes at P7 was confirmed in the GENSAT Database (Supplementary Figure 1). Of these genes, we focussed on the neuron-specific serine protease inhibitor (neuroserpin), which was initially identified as an axonally secreted protein from neuronal cultures of chicken dorsal root ganglia and belongs to a serine protease inhibitor (serpin) gene family (Osterwalder et al., 1996). To analyze the expression pattern of neuroserpin protein in postnatal and adult mouse brain, we performed immunohistochemical analysis (Figure 3). In the P8 mouse brain, neuroserpin was detected in layer 5 pyramidal cells and subplate neurons (Figure 3A) and co-localized with the ER stress marker BiP (Figures 3B–F). The co-localization of neuroserpin and BiP in these neurons suggests that the production and secretion of neuroserpin contributes to the ER stress condition during the postnatal period. In adult, on the other hand, we could not detect neuroserpin expression in subplate neurons. A selected population of pyramidal cells in layer 5 expresses large levels of neuroserpin also in the adult. This is further supported by our layer-specific transcriptomic analysis in the adult (Belgard et al., 2011; Hoerder-Suabedissen et al., 2013). Similarly, BiP was absent from the subplate but present in layer 5 pyramidal cells in adult brains (Figures 3G–I). These results strongly suggest that subplate neurons have a protein secretion function during the postnatal period, but not or much reduced in adulthood.

## Discussion

In this study, we present several lines of evidence that rodent subplate neurons have a protein secretion function in the early postnatal period: firstly, subplate neurons in P8 mouse brain have very rich Nissl substance with ovoid cell shape and a non-central distribution of the nucleus, similar to other cells of known secretion function. Secondly, signs of ER stress are present in subplate neurons at P8, similar to other dedicated secretory cells. Thirdly, our ultrastructural examination of P8 rat subplate neurons revealed highly developed rough ER, which filled a large part of the cytosol. Fourthly, some genes, whose products are known to be secreted into the extracellular space, are expressed at high levels in subplate neurons of the P8 mouse brain (Table 1) but not necessarily in adult brains. Finally, neuroserpin, one such secreted protein, is likely to be located in ER of subplate neurons at P8 in the mouse brain (Figure 3).

We have shown that rodent subplate neurons (during the early postnatal period) and plasma cells have three common features; non-central distribution of the nucleus, highly developed rough ER filling a large part of the cytosol, and signs of ER stress condition. The characteristic morphological features of plasma cells at the light and electron microscopic levels have been described in details in the literature (Bloom and Fawcett, 1968). These three common features prompt us to suggest that subplate neurons have a protein secretory function.

During the early postnatal period, subplate neurons are the only cortical cell type with these three properties. In contrast, layer 5, some layer 6 and 2–3 pyramidal cells fit just one condition—exhibiting ER stress hallmarks (Figures 1C,D,G,H). The rough ER of layer 5 pyramidal cells is restricted to the cytosol and the nuclei are located centrally within the cytosol (Parnavelas et al., 1978; Miller and Peters, 1981) in contrast with subplate (present study). Furthermore, the proportion of cytosol occupied by rough ER is much higher in subplate than in orther cortical neurons (data not presented). These results suggest that although several cell types may have secretory properties, subplate neurons may be more specialized to protein secretion than other cells. Interestingly, subplate neurons in adult stage have relatively small cytoplasm and are no longer under ER stress conditions (Figures 1F,H). This suggests that the secretory function of subplate neurons is transient.

Some genes, whose products are known to localize in the extracellular space, are very strongly expressed in subplate neurons in the P8 mouse brain (Table 1). Connective tissue growth factor (CTGF) belongs to a family of secreted, extracellular matrix-associated proteins that are involved in the regulation of cellular functions such as adhesion, migration, mitogenesis, differentiation and survival (Brigstock, 1999) as well as maturation of oligodendrocytes and progression of myelination (Stritt et al., 2009). We have previously reported that CTGF expression is detectable in the subplate region at E18 and increases in the number of cells and the intensity of labeling at P3 and P8 (Hoerder-Suabedissen et al., 2009, 2013). Neuroserpin is an inhibitor of tissue plasminogen activator (tPA) that is expressed in developing and adult nervous systems (Hastings et al., 1997; Krueger et al., 1997). Mutations in neuroserpin result in its misfolding and accumulation in the ER (Miranda et al., 2004). In this study, immunohistochemical analysis demonstrated that subplate neurons in P8 mouse brain express neuroserpin, which may be released by secretion. Neuronal pentraxin 1 (Nptx1), predicted to be a secreted protein, is selectively expressed in the nervous system and has been suggested to be involved in synaptic functions (Schlimgen et al., 1995; Dodds et al., 1997). Insulin-like growth factor binding protein 5 (IGFBP-5), which is an extracellular modulator of Insulin-like growth factor (IGF) signaling, has been highlighted as a focal regulatory factor during the development of several key cell lineages, e.g., myoblasts and neural cells (Clemmons, 1997; Cheng et al., 1999; Pera et al., 2001). GENSAT Database shows that the genes encoding these secreted proteins are expressed in the subplate region at P7 mouse brain (Supplementary Figure 1; Hoerder-Suabedissen et al., 2013). This period coincides with the major changes in somatodendritic morphology and death of subplate cell populations (Hoerder-Suabedissen and Molnár, 2012, 2013, 2015). To elucidate the function of subplate neurons during postnatal period, it may be useful to investigate the functions of these secreted proteins during normal development and in pathological conditions, including after perinatal hypoxic ischaemic brain damage (Okusa et al., 2014).

## Conclusion

Our work shows that during the postnatal period subplate neurons in rodents have highly developed rough ER, transiently express neuroserpin, a secreted protein and show signs of ER stress. Taken together, these results suggest a transient protein secretory function of rodent subplate neurons during the postnatal period.

### Conflict of interest statement

The authors declare that the research was conducted in the absence of any commercial or financial relationships that could be construed as a potential conflict of interest.

## Supplementary material

Supplementary Figure1 **Expression patterns of secretory protein genes in P7 sagittal mouse brain**. *In situ* hybridization images from the GENSAT NCBI website (http://www.ncbi.nlm.nih.gov/sites/entrez), numbers indicate references at the time of the download. **(A**) CTGF: (GENSAT Image 60754), **(B)** Neuroserpin/SERPINI1: (GENSAT Image 51784), **(C)** Nptx1: (GENSAT Image 18886), **(D)** IGFBP-5: (GENSAT Image 17034).

## Abbreviations

ER: endoplasmic reticulum
BiP: immunoglobulin heavy chain-binding protein
GRP78: 78 kDa glucose-regulated protein
CTGF: connective tissue growth factor
neuroserpin: neuron-specific serine protease inhibitor
Nptx1: Neuronal pentraxin 1
IGFBP-5: Insulin-like growth factor binding protein-5.
