# Supplementary figures and images for: Secretory function in subplate neurons during cortical development
Source: Front Neurosci. 2015 Mar 26;9:100. doi: 10.3389/fnins.2015.00100 (PMC4374456; doi:10.3389/fnins.2015.00100)

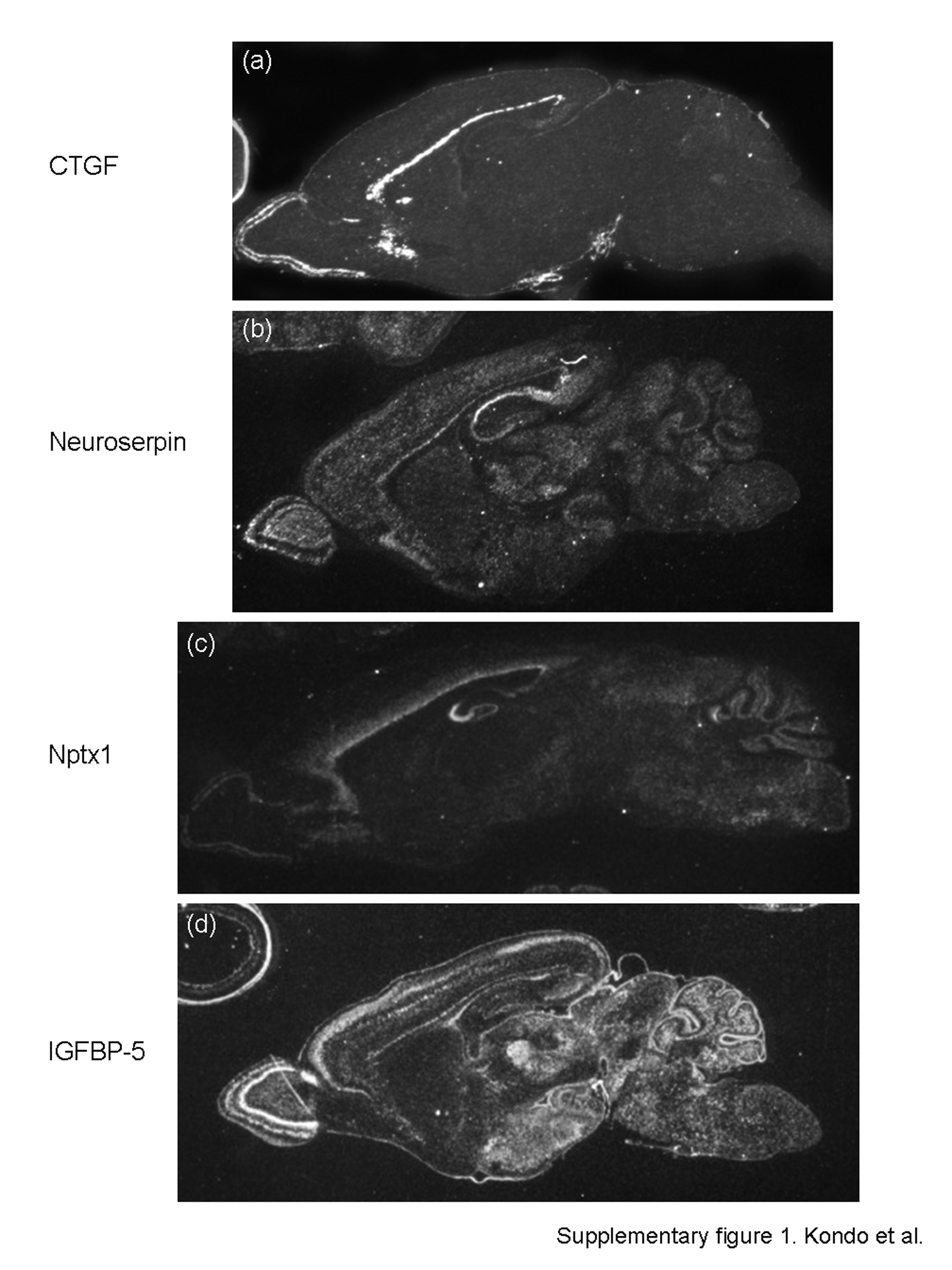

Supplement: Supplementary Figure1 — Expression patterns of secretory protein genes in P7 sagittal mouse brain. In situ hybridization images from the GENSAT NCBI website (http://www.ncbi.nlm.nih.gov/sites/entrez), numbers indicate references at the time of the download. (A) CTGF: (GENSAT Image 60754), (B) Neuroserpin/SERPINI1: (GENSAT Image 51784), (C) Nptx1: (GENSAT Image 18886), (D) IGFBP-5: (GENSAT Image 17034). [file Image1.TIF]
